# Study on the preparation and enzyme inhibitory activity of polyphenols from *Sargassum pallidum*

**DOI:** 10.1371/journal.pone.0297434

**Published:** 2024-01-30

**Authors:** Haiyun Jiang, Li Kong, Hongguang Tang, Zhenzhen Wang, Caiping Liu, Jianhui Zhang, Yuxin Chen, Jinyang Shen, Yue Zhou

**Affiliations:** 1 Department of Pharmacy, Jiangsu Ocean University, Lianyungang, Jiangsu, China; 2 Department of Pharmacy, Lianyungang Hospital of Traditional Chinese Medicine, Lianyungang, Jiangsu, China; Université du Québec à Trois-Rivières: Universite du Quebec a Trois-Rivieres, CANADA

## Abstract

This study aimed to obtain a high yield and purity of *Sargassum pallidum* polyphenol extracts (SPPE) and study its enzyme activity. Fresh *Sargassum pallidum* seaweed was selected for optimization of ultrasound-assisted extraction (UAE) conditions and purification conditions using macroporous resin and Sephadex LH20 to obtain SPPE. The SPPE was characterized using UPLC-QTOF-MS/MS and α-amylase, α-glucosidase, tyrosinase, and AchE inhibitory activity were determined. The maximum extraction rate of SPPE was 7.56 mg GAE/g and the polyphenol purity reached 70.5% after macroporous resin and Sephadex LH-20 purification. A total of 50 compounds were identified by UPLC-QTOF-MS/MS. The IC_50_ values of SPPE were 334.9 μg/mL, 6.290 μg /mL, 0.834 mg /mL and 0.6538 mg /mL for α-amylase, α-glucosidase, tyrosinase and AchE, respectively. Molecular docking technology further revealed the effects of SPPE on the above enzymes. This study provided information on the potential hypoglycemic, whitening and anti-Alzheimer’s disease biological activities of SPPE, which had guiding significance for the purification and development of other seaweed polyphenols.

## 1. Introduction

Seaweed is one of the most important organisms in the ocean, including brown, red and green algae, and is a good source of proteins, lipids, carbohydrates and other bioactive compounds [1–6]. It has been reported that phenolic compounds in seaweed have various pharmacological activities, including antioxidant, antibacterial, antiviral, anticancer, anti-inflammatory, and anti-diabetic [2, 4, 7]. These phenolic compounds have been exploited not only for their biological activity but also for their potential to prevent a wide range of chronic diseases such as cancer, cardiovascular disease, neurodegenerative disorders, obesity, and diabetes [2, 4, 8]. Due to their nutritional and health benefits, seaweed polyphenols are increasingly being investigated for possible use in nutritional medicines, functional foods, cosmetics, and pharmaceutical applications [9].

Seaweed polyphenols could be extracted by different methods. The most typical one was the conventional liquid extraction by maceration [10]. This involved the contact of the matrix with high volumes of solvents during long periods, at room or high temperatures [11]. As polyphenols were moderately polar compounds, methanol, ethanol, or their aqueous mixtures (e.g., 50% ethanol), high temperatures and long extraction times need to be used to increase yield [12]. In order to solve the shortcomings of traditional extraction methods, ultrasonic assisted extraction was adopted. By improving extraction efficiency, shortening extraction time, adjusting extraction parameters according to the characteristics of polyphenols, the selective extraction was realized, the structure of polyphenols was preserved, and the solvent residue was reduced [13]. And high yield of polyphenols could be achieved by ultrasound-assisted extraction technology [14]. This fraction was further fractionated by Sephadex LH-20 column chromatography or ultra-filtration [15].

It is a very challenging task to extract high purity polyphenols from seaweed. The purity of polyphenols extracted from seaweed is usually less than 42% [16–18]. In order to achieve a high degree of polyphenol purity, the primary techniques employed included liquid-liquid extraction and solid-phase extraction (SPE), which were selected based on the polarity of the molecules, along with dialysis, which was chosen based on the molecular size. Using liquid-liquid extraction with organic solvent could obtain enriched polyphenol fractions from raw extracts [11, 19–21]. The utilization of organic solvents, including ethyl acetate, poses a significant health risk due to its toxic nature and potential for causing organ damage through repeated inhalation or ingestion. Consequently, it is advisable to employ safer alternatives, such as microporous resins, silica gel, dispersants, and Sephadex LH-20, in SPE procedures [22].

The extracts from *Sargassum pallidum* showed antioxidant activity [23] and α-glucosidase inhibitory activity [24], respectively. Previous studies showed seaweed polyphenols could be used as angiotensin-converting enzyme (ACE) inhibitors [25, 26], matrix metalloproteinase (MMPs) inhibitors [27], acetylcholinesterase (AChE) and butyrylcholinesterase (BChE) inhibitors [28, 29], and tyrosinase inhibitors [30].

However, apart from *Sargassum pallidum* polysaccharides, limited research has been conducted on the polyphenolic constituents of *Sargassum pallidum*, with only Xu et al. [31] and Ye et al. [32] identifying flavonoid and phloroglucinol derivatives from *Sargassum pallidum*, respectively. Hence, this study systematically investigated the extraction, purification, and bioactivity of polyphenol extracts from *Sargassum pallidum* (SPPE) with the aim of obtaining SPPE with high yield and purity, and broadening the scope of *Sargassum pallidum*’s deep processing products. This study presented comprehensive findings regarding the potential hypoglycemic, whitening, and anti-Alzheimer’s properties of SPPE, which was beneficial for the isolation of related enzyme inhibitors and the development of related agents. At the same time, it provided a theoretical basis and technical guidance for the industrial development of polyphenol extraction and processing of *Sargassum pallidum*.

## 2. Materials and methods

### 2.1 Chemicals and materials

*Sargassum pallidum* was obtained from Weihai, China, in September 2021. It was identified as *Sargassum pallidum* by Jinyang Shen of Jiangsu Ocean University. Acarbose was purchased from the National Institutes for Food and Drug Control, China. α-amylase (from porcine pancreas) and α-glucosidase (from saccharomyces cerevisiae) were purchased from Sigma-Aldrich, USA. Tyrosinase was purchased from Shanghai Yuanye, China. Anhydrous ethanol, folinol, soluble starch and sodium hydroxide were purchased from Sinopsin Group Chemical Reagent Co., LTD., China. Gallic acid was purchased from Shanghai Aladdin Biochemical Technology Co., LTD., China. Anhydrous sodium carbonate was purchased from Tianjin Yongda Chemical Reagent Co., LTD., China. Methanol, dinitrosalicylic acid (DNS), 4-nitrobenzene-α-D-glucoside (PNG), glycerol, disodium hydrogen phosphate (Na2HPO4), and dipotassium hydrogen phosphate (K2HPO4) were all analytically pure and purchased from Shanghai Macklin Biochemical Co., Ltd., China. Tyrosinase (BR), L-Dopa, Kojic acid, acetylcholinesterase (derived from electric eel), thio-acetylcholine iodide (ATCI), Dithio-dinitroformic acid (DTNB), Donepiperazine hydrochloride were purchased from Shanghai Macklin Biochemical Co., Ltd., China. Chromatographic grade formic acid, acetonitrile and ammonium formate were purchased from Merck KGaA, Germany.

### 2.2 Preparation of *Sargassum pallidum* polyphenol extracts (SPPE)

#### 2.2.1 Extraction of crude extracts

*(1) Ultrasound-assisted extraction (UAE)*. *Sargassum pallidum* seaweed was selected for optimization of UAE conditions to obtain a high yield of phenolic compounds. Fresh *Sargassum pallidum* seaweed was washed, dried, and freeze-dried using a freeze-dryer for 40–50 h. It was crushed through a 60-mesh sieve, and stored in dry conditions away from light. UAE was conducted using an ultrasonic bath (Sumei Sonorex KQ2200DE, Kunshan, China). The frequency of the ultrasonic bath was fixed at 40 kHz, 500 W, and the algae powder samples were placed in a beaker with the solution [33]. The ultrasonic bath was carried out at the required temperature for needed extraction times. After ultrasonic extraction, the extract was filtered under negative pressure and the filtrate was collected. The filtrate was evaporated to remove the solvent. The extract was vacuum dried to obtain the crude extracts. The total phenolic content (TPC) of the extracts was determined according to the method under 2.3, and the polyphenol purification level was calculated using the following formula:

P(%)=100CV/M (1)

Where P is the polyphenol purification level (%); C is the TPC of the sample solution (mg GAE/mL); V is the sample solution volume (mL) and M is the sample weight (mg).

*(2) Extraction conditions optimization*. The optimum extraction conditions for SPPE were determined by single-factor tests and the Orthogonal Experimental method. First, the extraction process was performed by systematically varying one parameter at a time, namely, the concentration of ethanol (40%、50%、60%), extraction time (20、30、40、50、60, min), material/liquid ratio (1:30、1:40、1:50, g/mL), extraction temperature (50、60、70,°C), and number of extraction (1、2、3).

Following the analysis and comparison of the single-factor test results, the factors were selected as independent variables in Orthogonal Experimental Design. Each factor was assessed at three levels. The factor levels of these four variables were coded as -1 (low), 0 (middle), and +1 (high), respectively.

The solution was collected and concentrated in a rotary evaporator at 45°C to remove the ethanol, and then freeze-dried. The purified products were weighed and dissolved in distilled water to make sample solutions. The TPC of the sample was determined according to the method under 2.3, and the polyphenol purification level was calculated using Formula (1).

#### 2.2.2 Purification of crude extracts

*(1) Macroporous resin purification*. The target components for enrichment and purification were polyphenolic, and macroporous resin was proposed as the material for enrichment and purification. Based on the range of applications and properties of the resins, 10 macroporous resins with a suitable range of applications and different polarities were selected as alternative resins based on the nature of the polyphenolic components to be enriched in this experiment, as shown in Table 1.

**Table 1:** Physical properties of different macroporous resins.

Resins	Polarity	Surface area (m^2^/g)	Average Pore Diameter (nm)	Particle diameter (mm)
AB-8	weak	480~520	13~14	0.3~1.25
XDA-7	weak	≥450	10~15	0.3~1.25
BS-45	Moderately	≥500	9~10	0.3~1.25
NKA-9	polar	250~290	15~16.5	0.3~1.25
ADS-17	Moderately	90~150	25~30	0.3~1.25
ADS-21	Hydrophobic	≥700	25~28	0.3~1.25
HPD100	Hydrophobic	650~700	8.5~9.0	0.3~1.2
LS305A	Hydrophobic	≥700	9~10	0.3~1.25
DA201	polar	≥200	10~13	0.3~1.25
XAD16N	Hydrophobic	≥800	10	0.56–0.71

The effect of different parameters on the adsorption/desorption properties of the polyphenolic compounds of the extract was compared. Specifically, the effect of macroporous resin column diameter ratio, the volume fraction of impurity removal ethanol, the volume fraction of elution ethanol, adsorption flow rate, sample concentration, sample volume, standing time, water volume, desorption volume, and desorption flow rate on adsorption efficiency were investigated by single factor experiments.


$$Q_{a}=(C_{0}-C_{1})V_{1}/W$$ (2)

$$Ar=[(C_{0}-C_{1})/C_{0}]\times 100\%$$ (3)

$$Q_{d}=C_{2}V_{2}/W$$ (4)

$$Dr=Q_{d}/Qe\times 100\%$$ (5)

$$Tr=Q_{d}/C_{0}V_{1}\times 100\%$$ (6)

Where *Q*_*e*_ is the adsorption capacity at the given contact time (mg GAE/g dry resin). *Ar* is the adsorption ratio (%). *Q*_*d*_ is the adsorption capacity at the given contact time (mg GAE/g dry resin). *Dr* is the desorption ratio (%). *Tr* is the transfer ratio (%). *C*_1_ is the concentration (mg GAE/mL) of phenolic in the solution at the given contact time. *C*_0_ is the initial concentration (mg GAE/mL) of phenolic in the solution. *V*_1_ is the volume of the initial sample solution (mL), and W is the resin weight (g). *C*_2_ is the concentration (mg GAE/mL) of phenolic in the solution after adsorption. *V*_2_ is the volume of the sample solution after adsorption (mL).

The enrichment was obtained under optimal conditions for dynamic adsorption of macroporous resin. The eluent was collected and concentrated in a rotary evaporator at 45°C to remove the ethanol, and then freeze-dried.

The purified products were weighed and dissolved in distilled water to make sample solutions. The TPC of the sample solutions was determined according to the method under 2.3, and the polyphenol purification level was calculated using Formula (1).

*(2) Sephadex LH-20 purification*. The sample purified by macroporous resin was further refined using Sephadex LH-20. The sample water solutions were then subjected to Sephadex LH-20 column chromatography using a stepwise gradient with a water/ethanol solvent system (ranging from 1/0 to 1/1 to 0/1). Each fraction was collected and concentrated in a rotary evaporator at 45°C to remove the ethanol, and then freeze-dried [34]. The TPC for each fraction was determined according to the method under 2.3, and the polyphenol purification level was calculated using Formula (1).

### 2.3 Determination of total phenolic content (TPC)

TPC was determined by Folin-Ciocalteu technique [35]. Briefly, 1.0 mL of the diluted sample and 2.5 mL of 10% (v/v) Folin-Ciocalteu were put together, shaken well, and incubated for 5 min under room temperature and darkness. After completing the previous steps, 2 mL of 7.5% (w/v) Na_2_CO_3_ was incorporated, bringing the total volume to 10 mL. The absorbance was measured at 750 nm after 2 h of reaction. Gallic acid was selected as the standard substance. The TPC of extracts was expressed as a gallic acid equivalent (mg GAE/g). The polyphenol concentration in sample solution was calculated by linear equation with a constant solution volume of 1mL. The TPC was calculated as follows [36]:

TPC=CNV/m (7)

Where V is the volume of the sample solution (mL). N is the determined dilution. C is the polyphenols concentration calculated according to the standard curve, and m is the mass of the dried seaweed (g).

### 2.4 UPLC-QTOF-MS/MS analysis

The HPLC analysis of the purified extracts was performed on Waters system equipped with an automatic sample manager and a diode array detector. The chromatographic separation was achieved on a Waters Acquity UPLC HSS C18 (2.1mm×100mm, 1.8 μm), and the temperature of the column was set at 35°C. The mobile phases included 0.1% methanol and 5 mM ammonium formate in water (A) and Acetonitrile (B) with a flow rate of 0.3 mL/min. The injection volume was 2 μL. After optimization of the elution condition, the method was as follows: 0~1min, 2% B; 1~22 min, 2%~95% B; 22~26.4min, 95% B; 26.4~26.5 min, 92%~2%B; 26.5~30 min, 2% B.

The mass spectrometer Xevo G2-XS Q-TOF-MS/MS (Waters Corp., USA) containing a Quadrupole-TOF combined with an ESI source was set in positive ionization mode. Parameters of optimum source were set as follows: capillary voltage, 3.0 kV; source temperature, 100°C; desolventizing temperature, 250°C; cone voltage, 30 V. The mass spectra calibrated at a scan range of 100~1200 Da, and Masslynx 4.1 software was used to identify the compounds by comparing the MS/MS fragment, molecular weight, and formula according to the database.

### 2.5 Enzyme inhibition assay

#### 2.5.1 Inhibition effect on α-amylase and α- glucosidase activity

The inhibitory activity of α-amylase evaluation was evaluated using the method mentioned in [35] with slight modification. Various extract concentrations (12.5–1500 μg/mL, 100 μL) were incubated with amylase solution (0.5 mg/mL, 100 μL) in 20 mM sodium phosphate buffer (pH 6.9) for 10 min at 25°C. Then, a starch solution of 200 μL (1%, w/v) was added and incubated at 37°C for 10 min. Subsequently, 100 μL dinitrosalicylic acid (DNS) was added to stop the reaction. The mixture was then heated to 100°C for 5 minutes and cooled to room temperature. The reaction mixture was diluted with 10 mL of distilled water and the absorbance was measured at 540 nm. Instead of the sample, controls were prepared by replacing sample dilution with buffer solution. The positive control was acarbose and the concentration range was 12.5–200 μg/mL. IC_50_ was defined as 50% inhibition of α-amylase activity at sample concentration. Making use of the following formula, the inhibitory activity of α-amylase was calculated.


$$\%Inhibition=\frac{{A540}_{control}-{A540}_{sample}}{{A540}_{control}-{A540}_{block}}\times 100\%$$ (8)

Where A540_control_ is the absorbance of negative control product without inhibitor at 540nm. A540_sample_ is the absorbance of caraway extract or positive drug and enzyme reaction at 540nm. A540_block_ is the absorbance of blank control without enzyme at 405nm.

The α-glucosidase inhibition assay was a modification of the method previously described in [36]. A volume of 50 μL of sample extract was diluted with 50 μL of 0.1 M potassium phosphate buffer (pH 6.9). Then, 100 μL of 0.1 M potassium phosphate buffer (pH 6.9) containing a glucosidase solution (1.0 U/mL) was added to the mixture, which was then incubated in 96-well plates at 25°C for 10 minutes. After preincubation, 100 μL of 5 mM p-nitrophenyl-a-D-glucopyranoside solution in 0.1 M potassium phosphate buffer (pH 6.9) was added to each well at timed intervals.

The reaction mixtures were incubated at 25°C for 20 min. After incubation, the absorbance was measured at 405 nm using a microplate reader. Instead of the sample, controls were prepared by replacing sample dilution with buffer solution. The positive control was acarbose and the concentration range was 12.5–200 μg/mL. IC_50_ was defined as 50% inhibition of α-amylase activity at sample concentration. Making use of the following formula, the inhibitory activity of α-amylase was calculated.


$$\%Inhibition=\frac{{A405}_{control}-{A405}_{sample}}{{A405}_{control}-{A405}_{Block}}\times 100\%$$ (9)

Where A405_control_ is the absorbance of negative control product without inhibitor at 405nm. A405_sample_ is the absorbance of wormwood extract or positive drug reaction with enzyme at 405nm. A405_block_ is the absorbance of blank control without enzyme at 405nm.

#### 2.5.2 Inhibitory effect on tyrosinase activity

The activity of tyrosinase was determined by spectrophotometry using L-DOPA as the substrate [37]. 60 μL of 0.1 M phosphate buffer (pH 6.8) and 20 μL of 50 U/mL tyrosinase were pipetted into a 96-well plate. Then, various concentrations of the compounds were added and incubated for 10 min at room temperature. After incubation, 40 μL of 10 mM L-DOPA was added to each microwell and the reaction was initiated. The absorbance was measured at 475 nm using a microplate reader. The 50% inhibitory concentration (IC_50_) values of the compounds were calculated using the formula given. Kojic acid was used as the positive control. The percent inhibition of tyrosinase activity was calculated as follows:

$$\%Inhibition=\frac{\left(A-B)-(C-D\right)}{\left(A-B\right)}\times 100\%$$ (10)

Where A is the absorbance at 475 nm without the test sample. B is the absorbance at 475 nm without the test sample and enzyme. C is the absorbance at 475 nm with the test sample. D is the absorbance at 475 nm with the test simple but without enzyme.

#### 2.5.3 Inhibitory effect on acetylcholinesterase (AChE)

AChE inhibiting activity was tested by Ellman’s method [38]. Briefly, the different concentrations of the sample (0.05–1.0 mg/mL), AChE (0.2 U/mL) and DTNB (10 mM) were mixed in PBS buffer (0.1 M, pH 8.0). With the solution of 180 μL as described above per well, the 96-well plate was pre-incubated at 37°C for 15 min. At 25 min after the addition of substrate (ATCI, 10 mM), the hydrolysis of substrate catalyzed by AChE was measured at 412 nm. A control experiment was performed under the same conditions with the same volume of purified water instead of the sample. A block experiment was performed under the same conditions with the same volume of PBS buffer instead of AChE. Donepezil was used as a positive control and the concentration range was 0.01–0.5 mg/mL. The IC50 was defined as the sample concentration that resulted in a 50% inhibition of AChE activity. The assay was performed in triplicate. The percentage of enzyme inhibition was calculated as follows:

$$\%Inhibition=\frac{{A412}_{control}-{A412}_{sample}}{{A412}_{control}-{A412}_{block}}$$ (11)

### 2.6 Molecular docking

To investigate the detailed intermolecular interactions, the molecular operating environment (MOE 2015.10) software package was used to perform various steps involved in docking simulation. The docking study was performed following the steps described in earlier reports [39–42].

The structural data of the proteins including α-amylase (PDB ID:1HNY), α-glucosidase (PDB ID:3WY1), tyrosinase (PDB ID:2X9Y), acetylcholinesterase (PDB ID:2CMF) were obtained from the RCSB PDB (http://www.rcsb.org/). The protein structure was 3D corrected and protonated, and all of the water molecules were removed before the creation of the enzymic action zones. The active zones were found using the self-contained site or site finder in MOE.

The structures of the main compounds in SPPE and acarbose were prepared using the ChemDraw 2021 software and optimized using the MOE 2015.10 software with energy minimization.

Docking was performed on the ligands toward the target enzyme using the MOE 2015.10 software. Docking of ligands to amino acid residues (GLN, LYS, TRP, GLU, APS, HIS, ILE, LEU, VAL, PHE, ALA, ASN, THR, TYR, TRP, GLY) on the target enzyme was carried out by using the same software. The refinement module was set to induce fit and retain of first and second scorings were set to 20 and 10, respectively. The London dG binding free energy scoring was used to rank the docking poses. A more negative value reflects a stronger interaction. The docked conformation which had the highest score was selected to analyze the mode of binding. The output data, including energy biding (docking score, DS), linkage types, binding residues, distances, and the energy biding of each linkage, were recorded.

### 2.7 Statistical analysis

All results were expressed as the mean ± standard deviation. Statistical analysis was accomplished by SPSS software, and a t-test was used to assay the difference between groups. *p* <0.05 was considered as significant difference.

## 3. Result

### 3.1 Optimization of the extraction conditions

**Fig 1:**
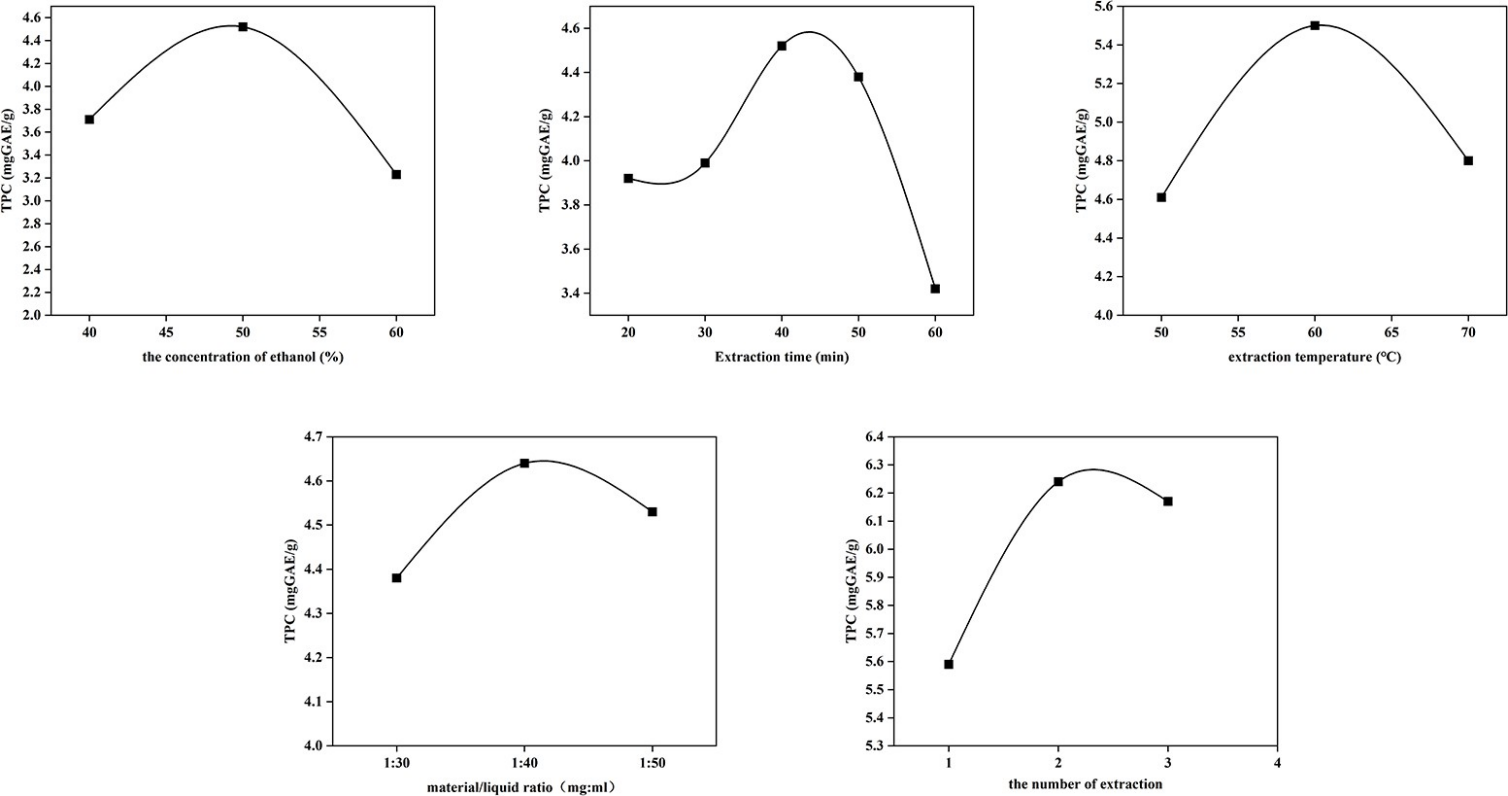
The effect on TPC (mgGAE/g) of different parameters.

The single-factor test result was shown in Fig 1. The optimal condition of ethanol concentration (50%), material/liquid ratio (1:40 g/mL), ultrasonic temperature (60°C), extraction time (40 min), and extraction times (twice) were determined based on single-factor tests. Under this condition, the TPC was 6.24 GAEmg/g.

Following the analysis and comparison of the single-factor test results, four factors were selected as independent variables in Orthogonal Experimental Design: concentration of ethanol (V: V, X1), material/liquid ratio (g/mL, X2), extraction temperature (°C, X3) and number of extraction (time, X4). Each factor was assessed at three levels.

The factor levels of these three variables were coded as -1(low), 0(middle), and +1(high) according to the single factor tests outlined below in Table 2. The Orthogonal Experimental group and TPC were shown in Table 3.

**Table 2:** Levels of independent variables.

Symbol	Independent Variables	levels		
		-1	0	+1
X1	concentration of ethanol (v:v)	45%	50%	55%
X2	material/liquid ratio (g/mL)	1:35	1:40	1:45
X3	extraction temperature (°C)	55	60	65
X4	number of extraction (time)	1	2	3

**Table 3:** The Orthogonal Experimental group and TPC (mgGAE/g).

	A	B	C	D	TPC (mgGAE/g)
1	1	1	1	1	4.70
2	1	2	2	2	6.38
3	1	3	3	3	6.81
4	2	1	3	2	7.57
5	2	2	1	3	5.86
6	2	3	2	1	5.38
7	3	1	2	3	6.64
8	3	2	3	1	4.98
9	3	3	1	2	5.53
K1	17.88	18.90	16.09	15.06	
K2	18.80	17.22	18.40	19.48	
K3	17.15	17.72	19.35	19.30	
k1	5.96	6.30	5.36	5.02	
k2	6.27	5.74	6.13	6.49	
k3	5.72	5.91	6.45	6.43	
R	0.55	0.56	1.09	1.47	

Under the optimal conditions of 50% ethanol concentration, 40 min extraction time, 65°C extraction temperature and two rounds of extraction, the maximum TPC was 7.57 mg GAE/g seaweed. In addition, the polyphenol purification level of the extracts under the optimal condition was 8.1%.

### 3.2 Preparative purification

#### 3.2.1 Macroporous resin purification

Adsorption/desorption properties of the polyphenolic compounds of the extract by the macroporous resins were shown in Table 4. The resin with the best adsorption/desorption performance was LS-305A resin. The adsorption and desorption rates were 66.5578% and 38.6991%, respectively. Therefore, LS-305A resin was chosen for a dynamic adsorption study.

**Table 4:** Adsorption/desorption properties on the polyphenolic compounds of the extract by the macroporous resins.

Resins	Q_a_(mg)	Ar(%)	*Q*_*d*_(mg)	*Dr*(%)
AB-8	1.628273	58.9369	0.501106	30.7753
XDA-7	1.718507	62.203	0.517031	30.0861
BS-45	1.743277	63.0996	0.593117	34.0231
NKA-9	1.621196	58.6808	0.534725	32.9834
ADS-17	1.68666	61.0503	0.522339	30.9689
ADS-21	1.642427	59.4492	0.465717	28.3554
HPD100	1.640658	59.3852	0.495798	30.2194
LS-305A	1.838818	66.5578	0.711607	38.6991
DA201	1.644197	59.5133	0.552371	33.5952
ADS16	1.661890	60.1537	0.516985	31.1083

A sample solution (TPC 0.48 GAE mg/mL, 0.1g seaweed/mL) was injected into the LS-305A resin column with a diameter column ratio of 1:4.29 at a speed of 1.0 BV/h, and with a bed volume of 60 mL. The polyphenol leakage ratio was 10% when the injection volume was 0.5 BV. This indicated that the dynamic adsorption equilibrium had been reached. The amount of elution water reached 70mL, and the anthrone sulfate reaction of elution water was negative, which proved that the impurities of the reduced sugar had been completely eluted. Therefore, the washing volume was set to 70mL in this experiment. The polyphenol elution rate was 20% at this point.

The 0.5 BV sample solution could be dynamically adsorbed by LS-305A resin and the adsorption capacity reached 14.5 mg GAE/g. The saturated resin was eluted with 50% (v/v) ethanol solution at 2.0 BV/h. The desorbed polyphenols were mainly concentrated in the 0~2.5 BV effluent solutions. The desorption equilibrium occurred when the resin was eluted with 2.5 BV ethanol solution with a polyphenol transfer ratio of 50%. The TPC of the extract was 23.5%.

#### 3.2.2 Sephadex LH-20 purification

The dried extract (1 g) was subjected to column chromatography on a Sephadex LH-20 gel and eluted in a water-ethanol (1:1) mixed solvent system. The TPC of the final product was 70.5%.

### 3.3 Chemical composition information of SPPE

**Fig 2:**
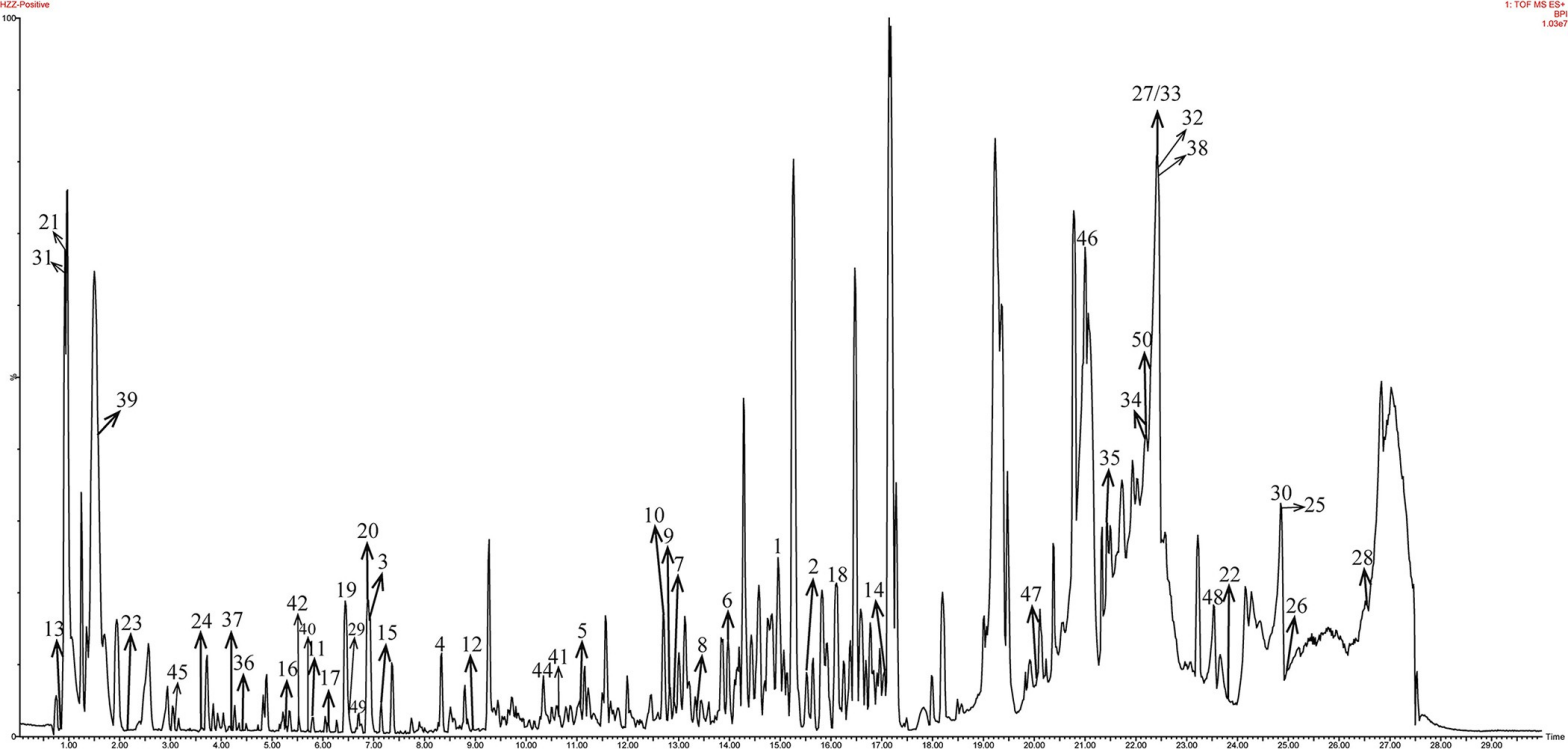
Total ion chromatogram of SPPE.

The total ion chromatogram of SPPE was shown in Fig 2. The information about the chemical composition of extracts in the database of the Phenol Explorer system and literatures were collected to establish a seaweed chemical composition database and imported into UNIFI 2.0 software. The mass spectrum data collected in the positive ion mode were automatically matched with the databases in UNIFI and QI software. Compounds with a deviation of less than 20 mDa were selected, identified and verified. The final results were shown in Table 5.

**Table 5:** Retention time, [M-H] +data and MS/MS fragmentation pattern for proposed compound 1 to 52 from SPPE.

Peak	Proposed compounds	RT (min)	molecular	weight	[M-H]+	MS/MS fragmentation pattern	classification
1	Carnosic acid	11.09	C_20_H_28_O_4_	332.1988	333.2047	237.10599[M-C7H11]; 255.10601[M-OH-CH3-C3H7]	organic acids
2	Carnosic acid/isomer	13.99	C_20_H_28_O_4_	332.1988	333.2045	317.17067[M-CH3]; 247.09607[M-C6H13]; 275.20089[M-COOH-CH3]	organic acids
3	Carnosic acid/isomer	12.91	C_20_H_28_O_4_	332.1988	333.2043	299.15956[M-OH-CH3]; 317.17067[M-CH3]	organic acids
4	Dihydrocaffeic acid	4.42	C_9_H_10_O_4_	182.0577	183.065	136.0621[M-COOH]; 167.0797[M-O]	organic acids
5	o-Coumaric acid	1.58	C_9_H_8_O_3_	164.0475	182.0802	130.049[M-2OH]; 147.0756[M-OH]	organic acids
6	3,4-DHPEA-AC	5.71	C_10_H_12_O_4_	196.0734	197.0807	147.0763[M-OCH3]	organic acids
7	4-Hydroxy-(3’,4’-dihydroxyphenyl)valeric acid	7.85	C_11_H_14_O_5_	226.0826	249.0736	152.062[M-CH2CH2COOH]; 201.0887[M-OH-OCH3]; 167.0798[M-CH2COOH]	organic acids
8	Carnosol	13.37	C_20_H_26_O_4_	330.1831	331.1889	315.1542[M-CH3]	phenols
9	Carnosol/isomer	12.85	C_20_H_26_O_4_	330.1831	331.1885	287.12224[M-C3H7]; 235.09346[M-C3H7-C4H4]	phenols
10	Carnosol/isomer	12.71	C_20_H_26_O_4_	330.1831	331.1886	315.1542[M-CH3]; 297.14322[M-CH3-OH]	phenols
11	(-)-Epicatechin	6.45	C_15_H_14_O_6_	290.079	313.0862	249.07046[M-C2HO]; 163.03633[M-OH-C6H6O2]; 181.05253[M-C6H5O2]	phenols
12	(+)-Catechin	6.89	C_15_H_14_O_6_	290.079	313.0863	249.07046[M-C2HO]; 277.11288[M-O]	phenols
13	(+)-Gallocatechin	0.94	C_15_H_14_O_7_	306.074	329.0573	263.07252[M-C2H3O]; 169.05781[M-C7H5O3]; 151.04665[M-C7H7O4]	phenols
14	3’-Hydroxy-3,4,5,4’-tetramethoxystilbene	3.62	C_17_H_18_O_5_	302.1154	325.113	163.05929[M-C7H6O2-OH]; 289.09136[M-CH3]; 145.0489[M-C7H6O2-2OH]	phenols
15	Carvacrol	10.34	C_10_H_14_O	150.1042	173.1232	131.0841[M-OH]; 141.0697[M-OH-CH3]; 159.0806[M-CH3]	phenols
16	Triphlorethol	12.20	C_18_H_14_O_9_	374.3010	375.2136	357.2061[M-H_2_0 + H]; 299.1995, [M-C_2_H_4_O_3_+ H]	phenols
17	Tetraphlorethol	4.72	C_24_H_18_O_12_	498.0798	499.2510	409.0772 [M-(H_2_0)_5_ + H]; 158[M-(H_2_0)_5_-C_12_H_11_O_6_ + H]	phenols
18	Gallic acid 3-O-gallate	5.8	C_14_H_10_O_9_	322.0325	323.0395	277.03404[M-COOH]; 305.02877[M-OH]; 261.0398[M-OH-COOH]	esters
19	Gallic acid ethyl ester	8.93	C_9_H_10_O_5_	198.0528	221.0418	169.00851[M-C2H5]; 153.0156[M-OC2H5]	esters
20	Gallic acid ethyl ester/isomer	0.88	C_9_H_10_O_5_	198.0528	221.0417	184.03784[M-CH3]; 149.99243[M-OH-C2H5]; 170.02411[M-C2H5]	esters
21	p-Coumaroyl glycolic acid	22.43	C_11_H_10_O_5_	222.0528	223.0635	149.04571[M-OCH2COOH]	esters
22	p-Coumaroyl glycolic acid/isomer	21	C_11_H_10_O_5_	222.0528	223.0633	149.04461[M-OCH2COOH]; 205.05337[M-OH]	esters
23	p-Coumaroyl glucose	5.28	C_15_H_18_O_8_	326.1002	327.1078	165.01634[M-2OH-CH2OH-C6H8O]; 179.03409[M-3OH-C6H8O]	sugars
24	1,2,2’-Triferuloylgentiobiose	23.79	C_42_H_46_O_20_	870.2582	893.2302	339.0988[M-OGlc-C20H16O6]; 308.1144[M-2C10H9O4-C10H8O3]	sugars
25	Luteolin 7-O-(2-apiosyl-6-malonyl)-glucoside	16.99	C_29_H_30_O_18_	666.1432	667.1525	550.12187[M-CH2COOCH2COOH]; 589.12124[M-CH2COOH-OH]; 518.12577[M-C8H4O3]	flavonoids
26	Neohesperidin	7.15	C_28_H_34_O_15_	610.1898	611.1981	303.0863[M-Rha-Glc]; 413.08179[M-Rha-OH-CH2OH]	flavonoids
27	p-Coumaroyl tartaric acid	6.11	C_13_H_12_O_8_	296.0532	297.0608	235.06015[M-OH-COOH]; 279.0503[M-OH]	flavonoids
28	2’,7-Dihydroxy-4’,5’-dimethoxyisoflavone	2.14	C_17_H_14_O_6_	314.079	315.0764	152.05631[M-C9H6O3]; 135.02962[M-C9H6O3-OH]	flavonoids
29	Daidzin 4’-O-glucuronide	24.96	C_27_H_28_O_15_	592.1428	593.1586	429.08916[M-Glc]; 251.04731[M-Glc-GlcA]; 371.10203[M-GlcA-CH2OH-OH]	flavonoids
30	Daidzin 4’-O-glucuronide/isomer	22.43	C_27_H_28_O_15_	592.1428	593.1583	265.0198[M-OGlcA-CH2OH-C8H7]; 223.06612[M-OGlc-COOH-C8H5O2]	flavonoids
31	Daidzein 7-O-glucuronide	26.68	C_21_H_18_O_10_	430.09	431.0867	371.10163[M-COOH-OH]; 147.06485[M-2OH-C15H5O4]	flavonoids
32	Formononetin 7-O-glucuronide	24.85	C_22_H_20_O_10_	444.1057	445.1203	281.05108[M-CH3-C5H8O5]; 355.07[M-C4H5O3]	flavonoids
33	Neohesperidin/isomer	0.91	C_28_H_34_O_15_	610.1898	633.1742	447.15635[M-OH-C9H6O2]; 463.13064[M-Rha]; 297.11639[M-CH2OH-Rha-C7H5O2]	flavonoids
34	Quercetin 3-O-galactoside 7-O-rhamnoside/	22.42	C_27_H_30_O_16_	610.1534	633.1507	281.05119[M-OGlc-OH-C8H5O2]; 355.07003[M-Rha-C6H4O2]	flavonoids
35	Quercetin 3-O-galactoside 7-O-rhamnoside/isomer	22.19	C_27_H_30_O_16_	610.1534	633.1508	355.06989[M-Rha-C6H4O2]; 281.05105[M-OGlc-OH-C8H5O2]	flavonoids
36	Quercetin 3-O-galactoside 7-O-rhamnoside/isomer	21.94	C_27_H_30_O_16_	610.1534	633.1502	385.14613[M-OGlc-3OH]	flavonoids
37	Quercetin 3-O-galactoside 7-O-rhamnoside	22.44	C_27_H_30_O_16_	610.1623	628.1955	266.9997[M-Rha-OGlc-OH]; 147.0649[M-C21H19O12]	flavonoids
38	Quercetin 3-O-glucuronide	10.66	C_21_H_18_O_13_	478.0708	479.0781	187.0025[M-C16H10O7]; 214.9975[M-C12H13O8]	flavonoids
39	Daidzein 7-O-glucuronide	5.51	C_21_H_18_O_10_	430.0918	431.099	236.0618[M-GlcA-OH]; 407.0364[M-OH]; 281.042[M-C8H5O3]	flavonoids
40	Luteolin 7-O-(2-apiosyl-6-malonyl)-glucoside/isomer	20.58	C_29_H_30_O_18_	666.1432	667.1702	559.13128[M-C6H3O2]	flavonoids
41	Formononetin 7-O-glucuronide/isomer	23.53	C_22_H_20_O_10_	444.1057	445.12	147.06467[M-2OH-C16H7O4]	flavonoids
42	Ellagic acid arabinoside/isomer	6.7	C_19_H_14_O_12_	434.0485	457.0563	241.08424[M-C8HO6]	flavonoids
43	Daidzin 4’-O-glucuronide/isomer	22.2	C_27_H_28_O_15_	592.1428	593.1586	355.06989[M-GlcA-C2H4O2]	flavonoids
44	24-Methyllathosterol ferulate	14.96	C_38_H_56_O_4_	576.4179	577.4227	315.16378[M-C19H33]; 339.16229[M-C17H33]	triterpenoids
45	24-Methyllathosterol ferulate/ isomer	15.51	C_38_H_56_O_4_	576.4179	577.4254	403.19525[M-C12H29]	triterpenoids
46	Arctigenin	6.94	C_21_H_24_O_6_	372.1573	373.164	355.15334[M-OH]; 235.10392[M-C_8_H_9_O_2_]	others
47	Arctigenin/isomer	8.34	C_21_H_24_O_6_	372.1573	373.1621	203.03177[M-C_8_H_9_O_2_-2OH]; 295.12953[M-C_2_H_5_O_3_]	others
48	Pinoresinol	16.11	C_20_H_22_O_6_	358.1416	359.1525	331.15692[M-CO]; 218.06254[M-C8H12O2]	others
49	6’’-O-Malonyldaidzin	24.85	C_24_H_22_O_12_	502.1111	503.108	281.05108[M-C8H13O7]	others
50	Ellagic acid arabinoside	6.51	C_19_H_14_O_12_	434.0485	457.0566	393.03769[M-C2HO]; 365.02725[M-4OH]	others
51	Caffeic acid ethyl ester	4.21	C_11_H_12_O_4_	208.0722	226.1053	168.0678[M-CH2CO]; 179.0812[M-CO]	others
52	4-Vinylsyringol	3.09	C_15_H_14_O_3_	242.0902	265.0797	153.0223[M-OH-C6H6O]; 195.0761[M-CH4O2]	others

A total of 52 compounds were identified in SPPE, including 7 organic acids, 5 esters, 10 phenols, 2 sugars, 19 flavonoids, 2 triterpenoids, and 7 others. Detailed compound information and MS/MS fragmentation pattern was shown in Table 5.

### 3.4 Enzyme inhibition assay

#### 3.4.1 Inhibitory effect of SPPE on α-amylase and α-glucosidase

**Fig 3:**
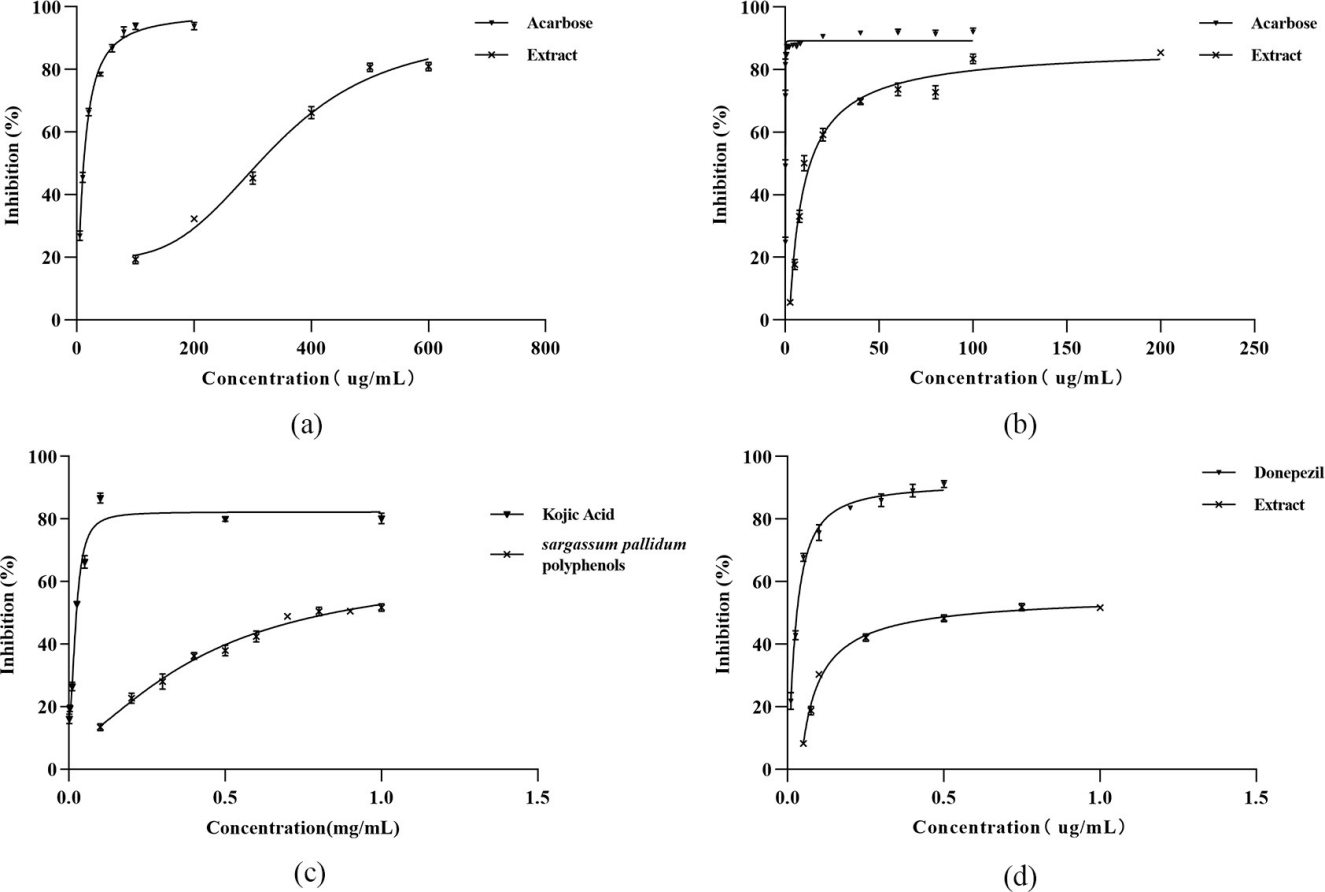
Percentage inhibition of SPPE. (a) α-amylase activity, (b) α-glucosidase activity, (c) tyrosinase activity, (d) AChE activity.

The percentage inhibition of α-amylase and α-glucosidase activity by SPPE and acarbose were presented in Fig 3A and 3B. SPPE showed α-amylase and α-glucosidase inhibitory activity with IC_50_ values of 334.9 μg/mL and 6.290 μg/mL, respectively. The positive drug showed stronger inhibitory effects with IC_50_ of 12.22 μg/mL and 0.02466 μg/mL, respectively.

#### 3.4.2 Inhibitory effect of SPPE on tyrosinase activity

The percentage inhibition of tyrosinase activity by SPPE and kojic acid were presented in Fig 3C. SPPE and the positive drug showed tyrosinase inhibitory activity with IC_50_ values of 0.834 mg/mL and 0.02097 mg/mL, respectively.

#### 3.4.3 Inhibitory effect of SPPE on AChE activity

The percentage inhibition of AchE activity by SPPE and donepezil were presented in Fig 3D. SPPE and donepezil showed AchE inhibitory activity with IC_50_ values of 0.6538 mg/mL and 0.03126 mg/mL, respectively.

### 3.5 Molecular docking

**Fig 4:**
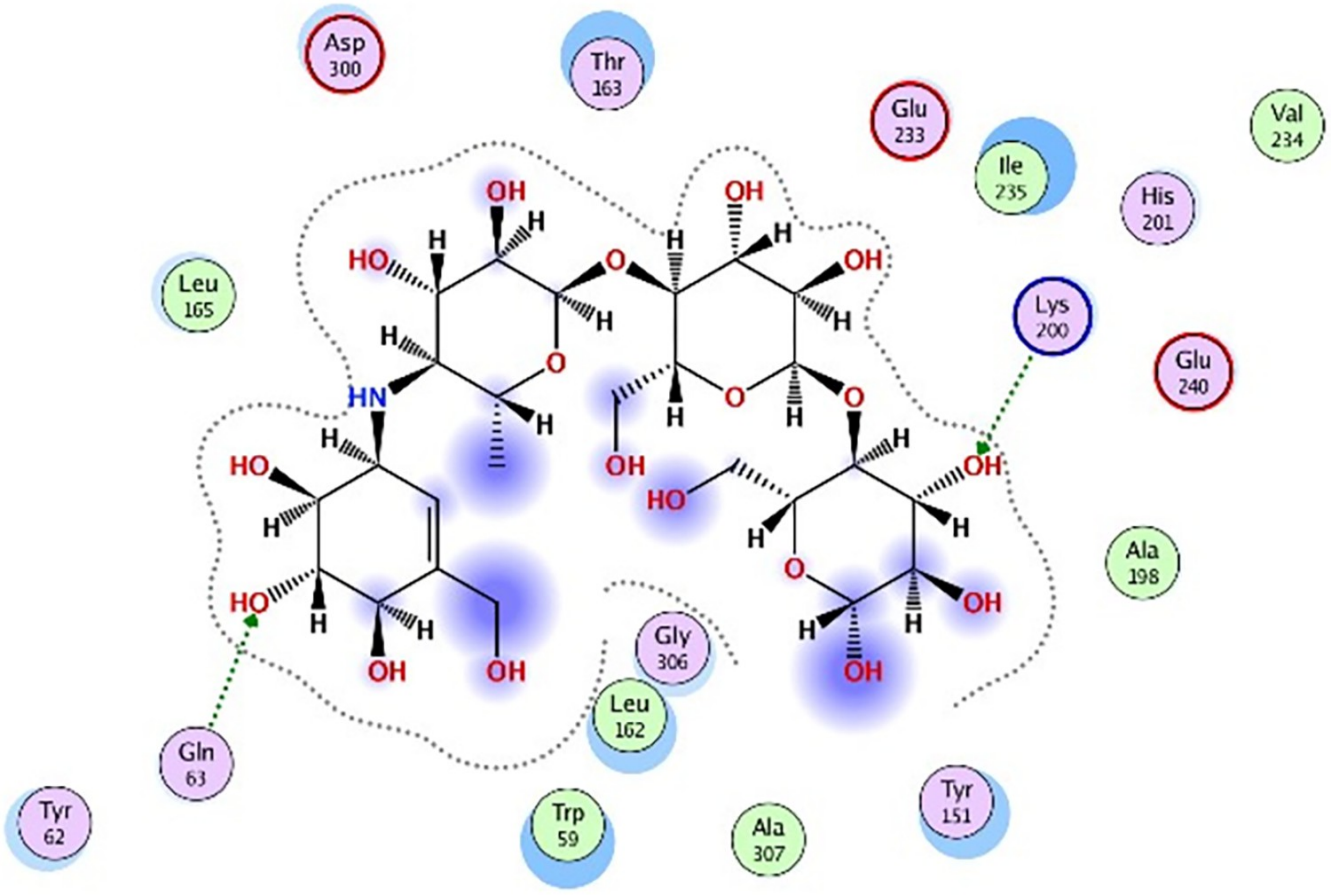
The docked binding mode of acarbose with α-amylase.

**Fig 5:**
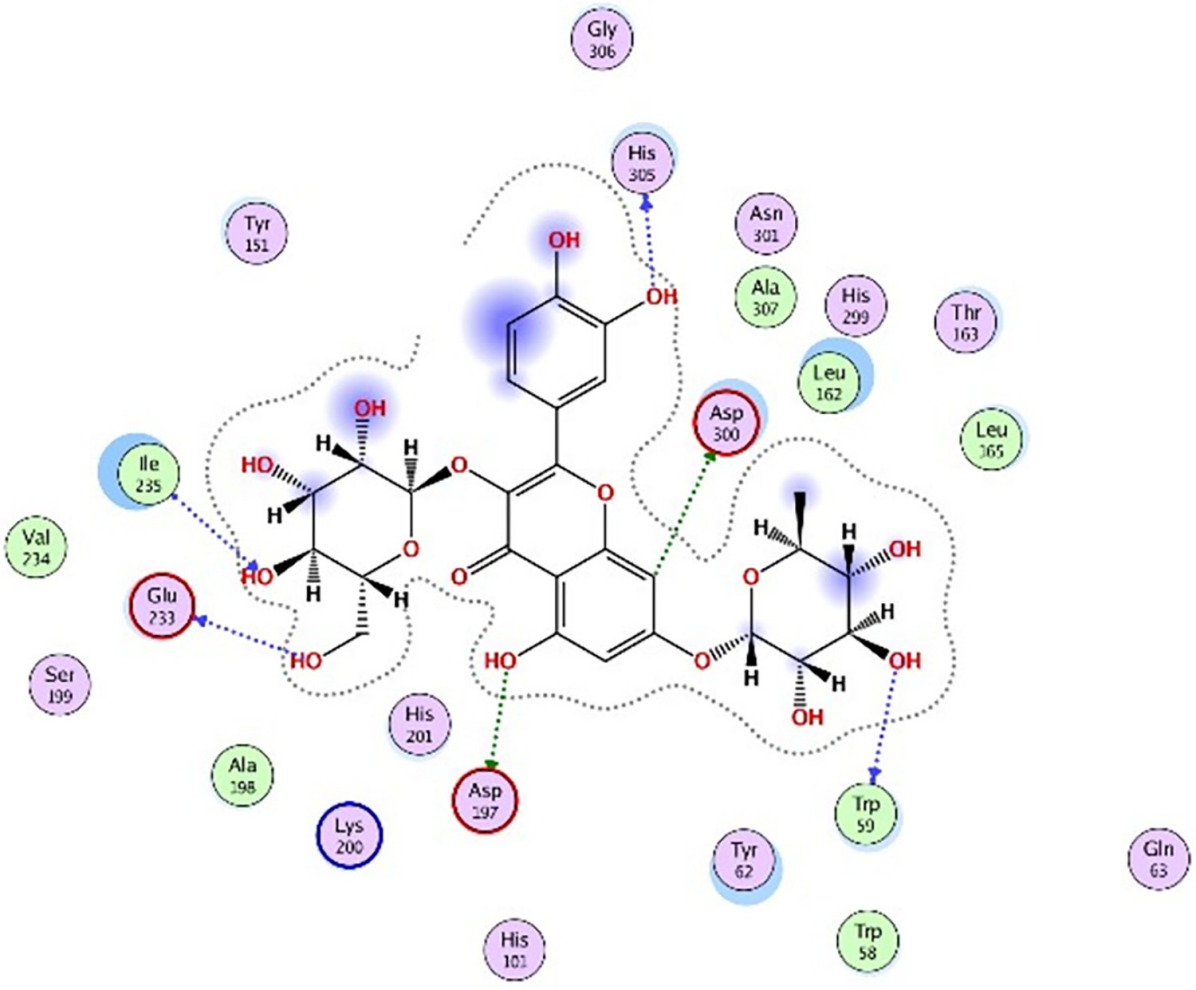
The docked binding mode of Quercetin 3-O-galactoside 7-O-rhamnoside with α-amylase.

**Fig 6:**
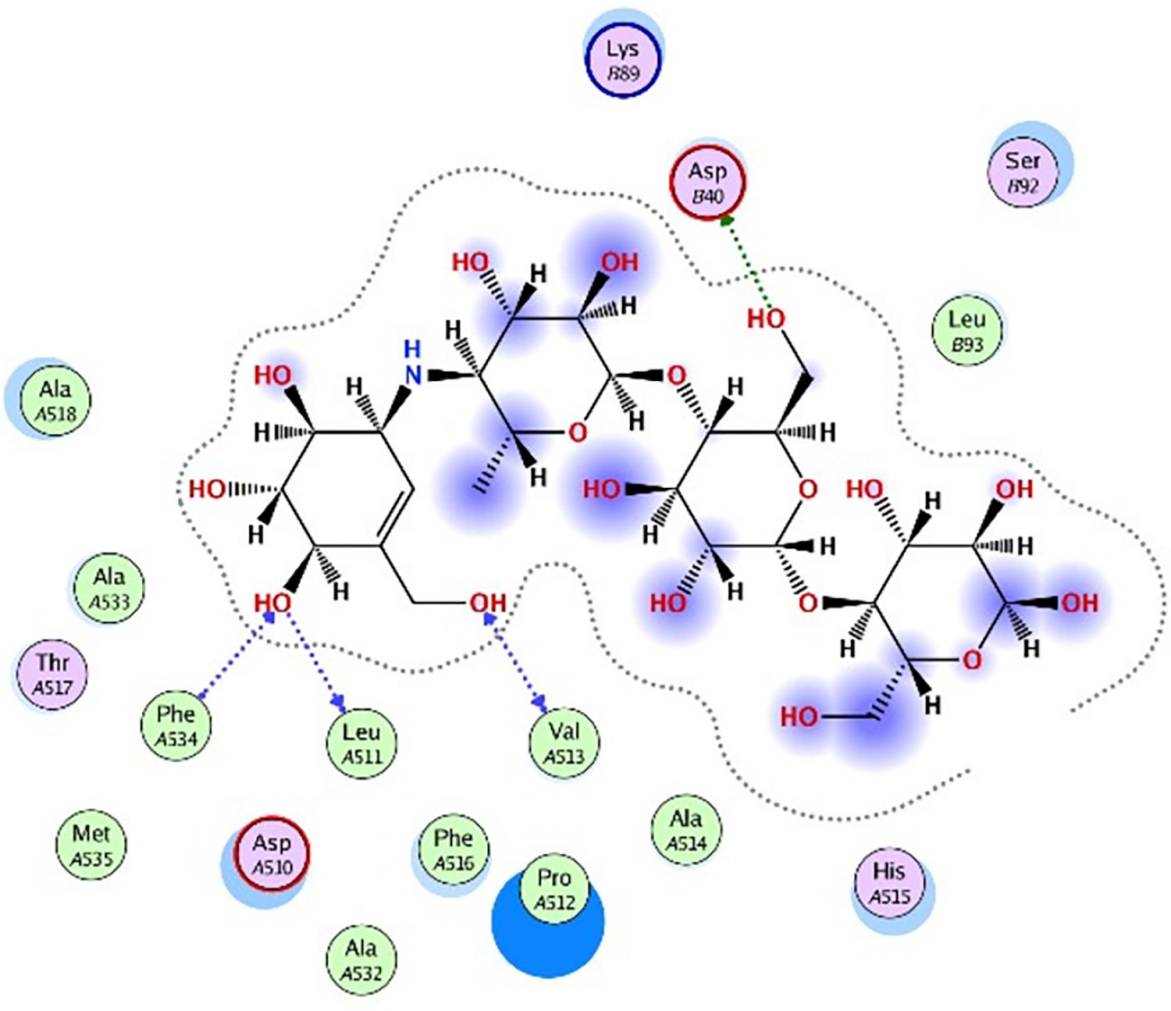
The docked binding mode of acarbose with α-glucosidase.

**Fig 7:**
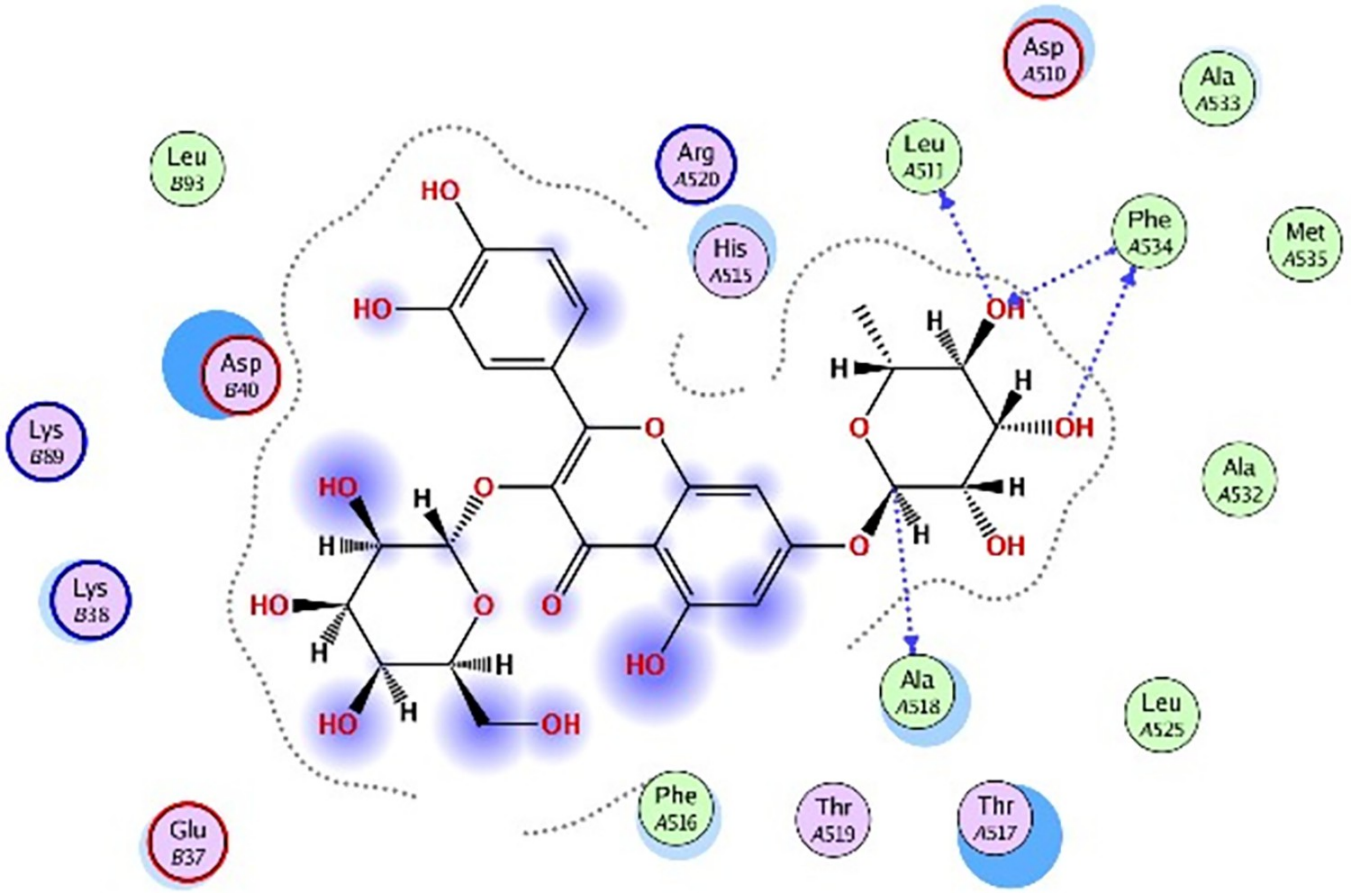
The docked binding mode of Quercetin 3-O-galactoside 7-O-rhamnoside with α-glucosidase.

**Fig 8:**
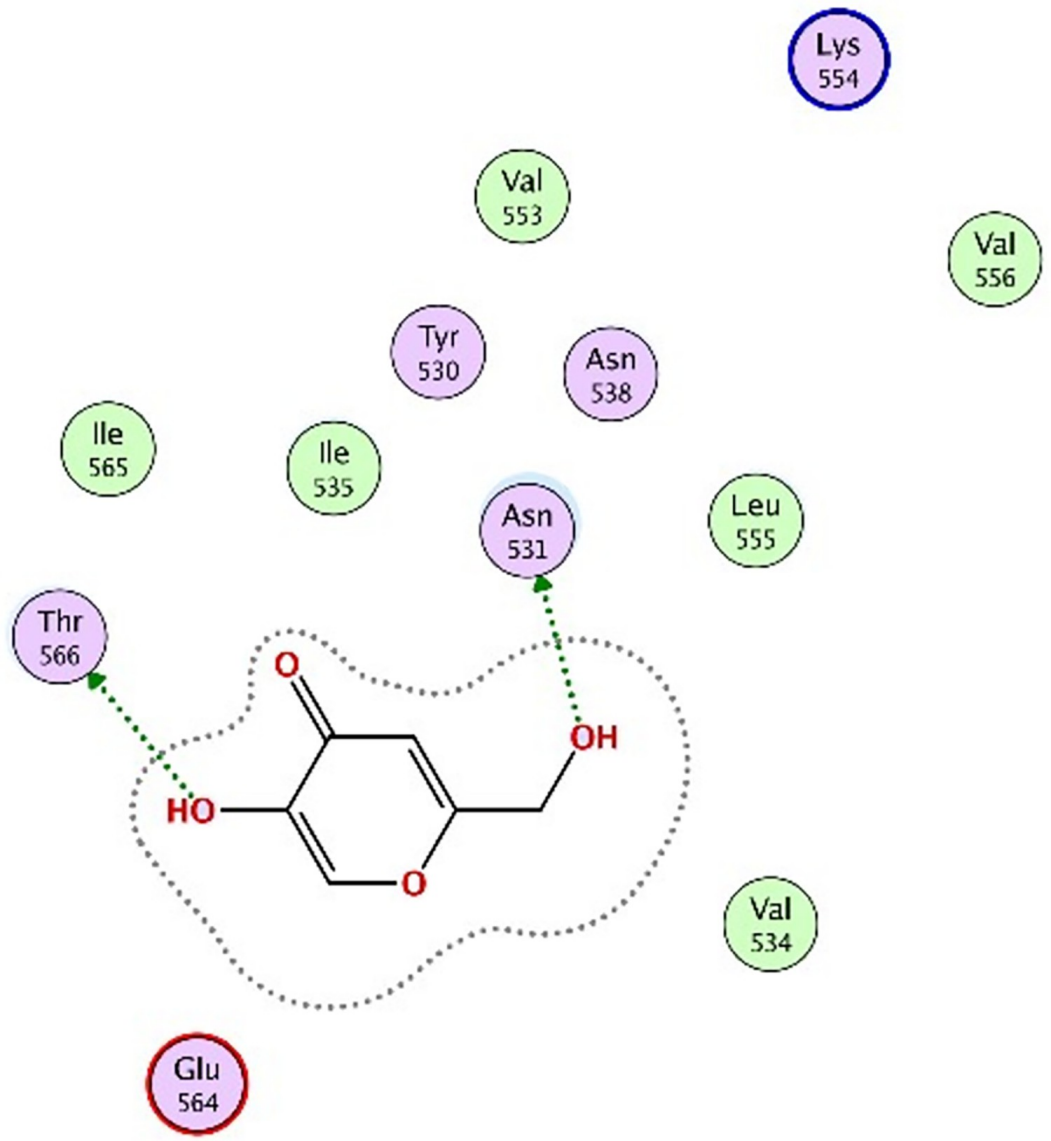
The docked binding mode of kojic acid with tyrosinase.

**Fig 9:**
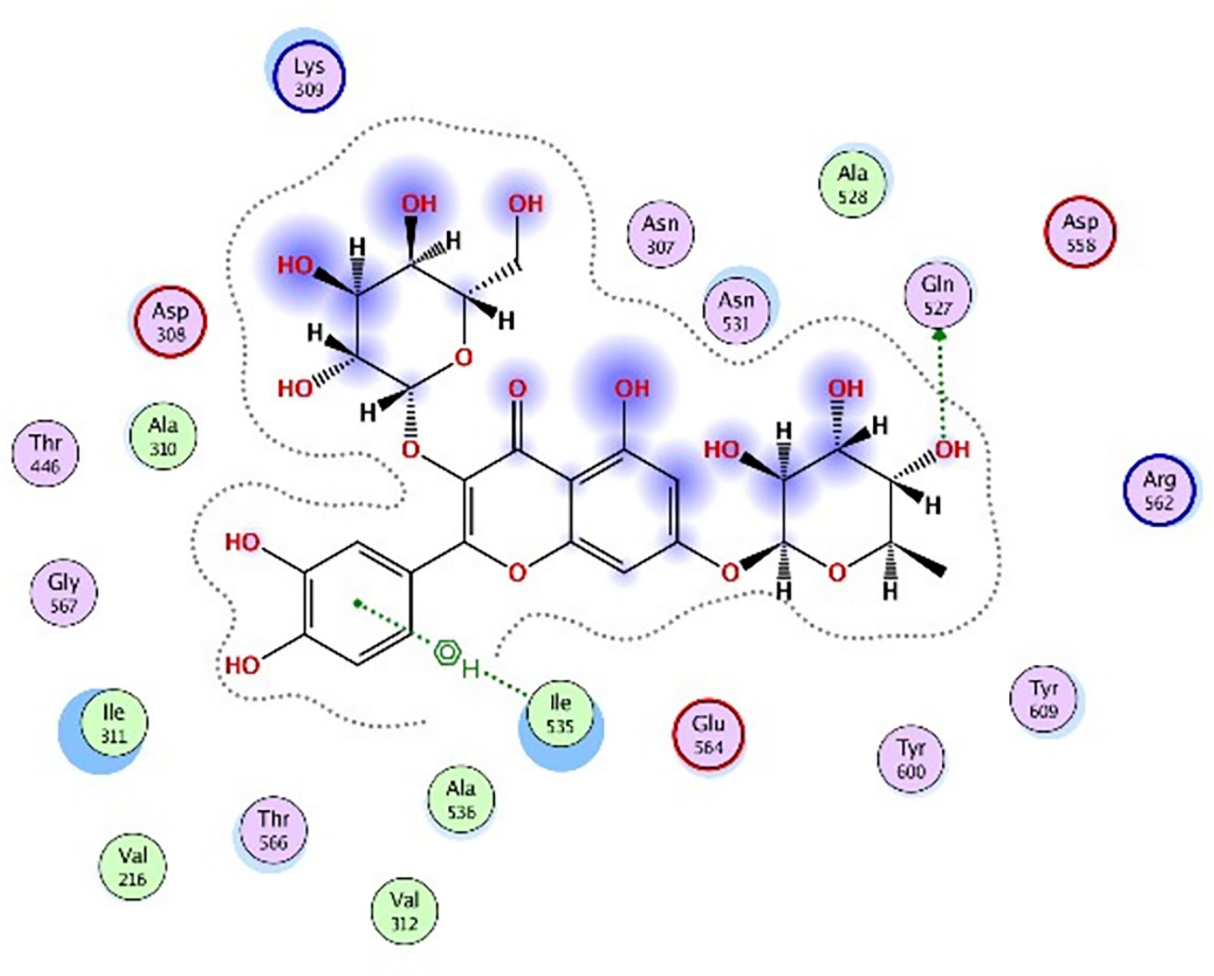
The docked binding mode of Quercetin 3-O-galactoside 7-O-rhamnoside with tyrosinase.

**Fig 10:**
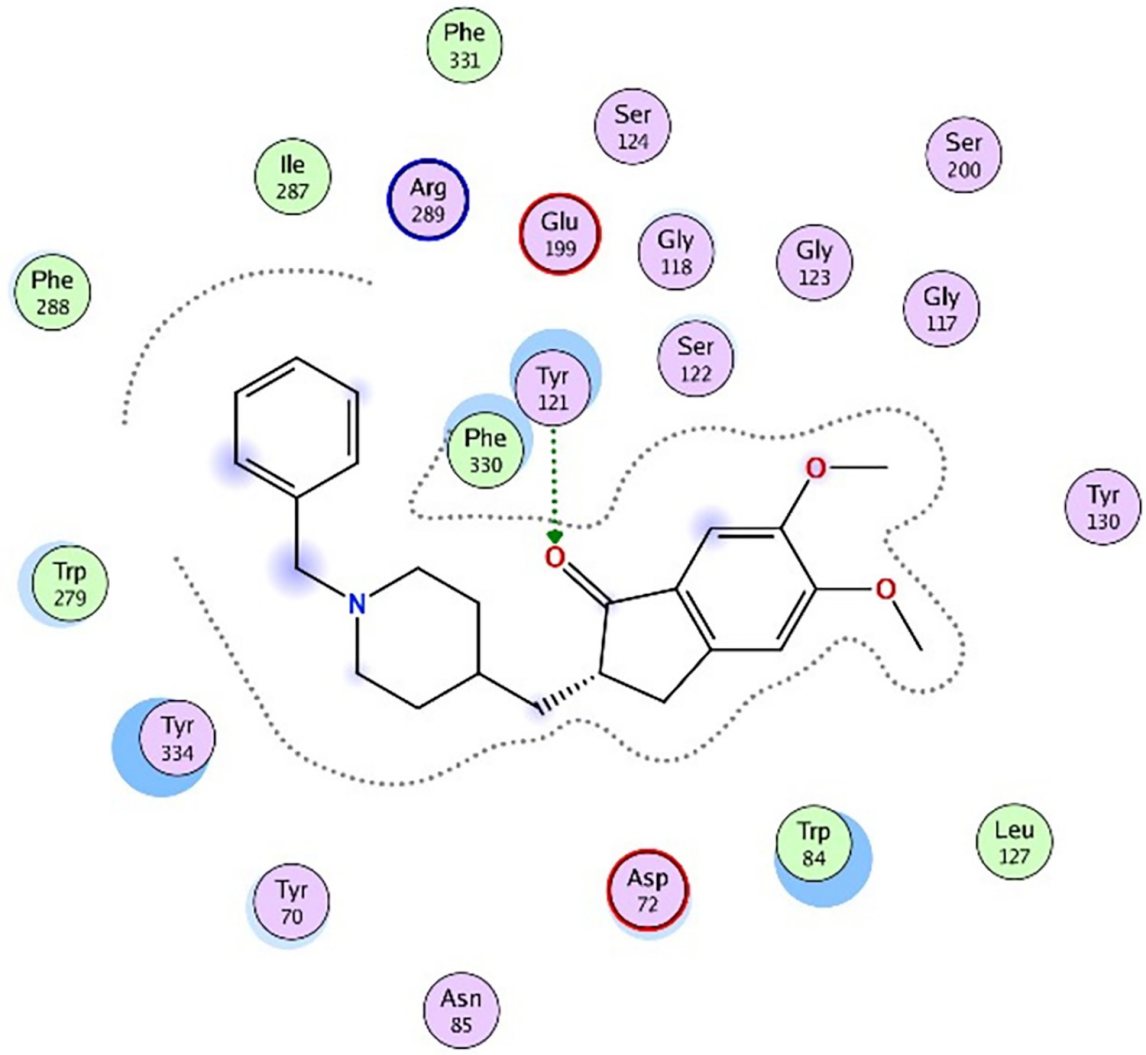
The docked binding mode of donepzil with AChE.

**Fig 11:**
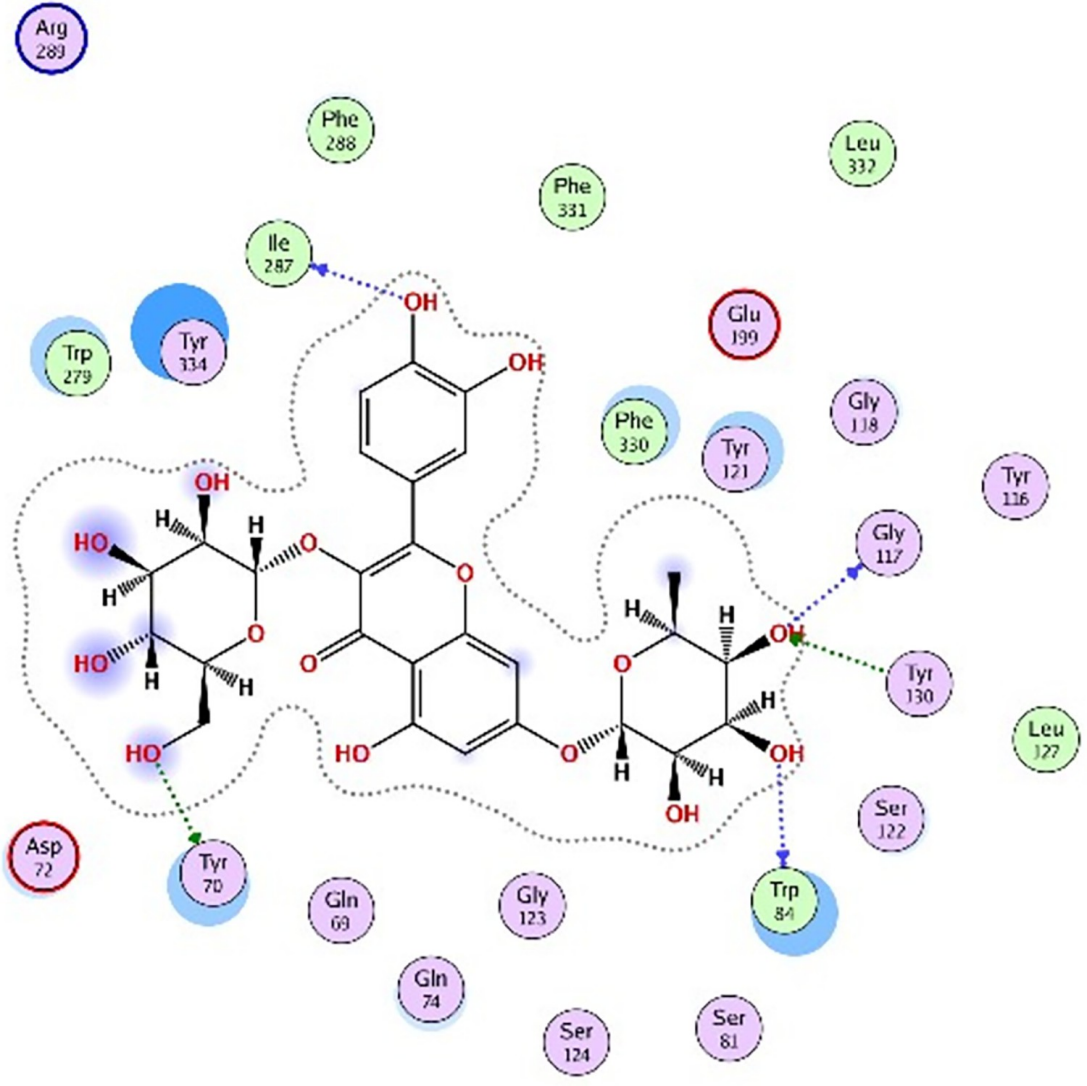
The docked binding mode of Quercetin 3-O-galactoside 7-O-rhamnoside with AChE.

**Fig 12:**
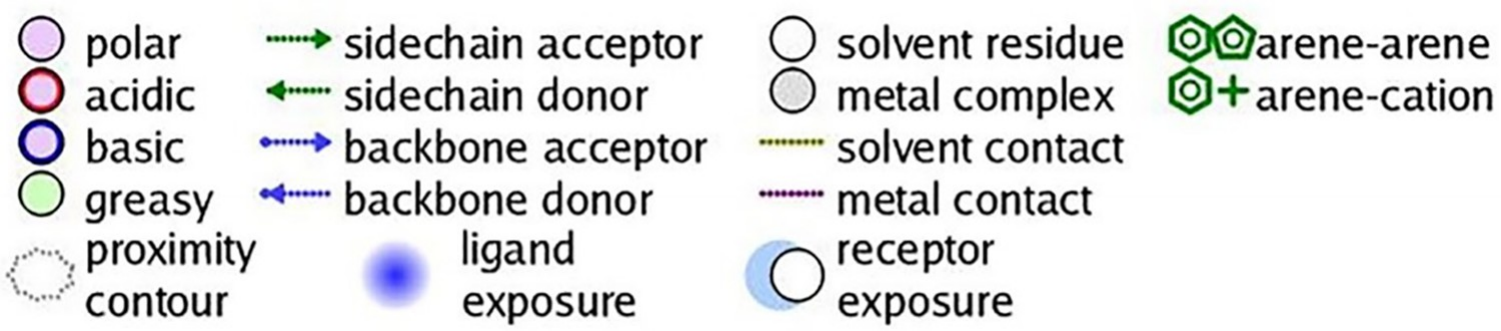
The annotation of molecule-target interactions in Figs 4 to 11.

Molecular docking was further carried out to better understand the action mechanism of SPPE on the above enzymes. The lower the score of the conformation, the more stable it is. Figs 4–11 displayed the conformation of Quercetin 3-O-galactoside 7-O-rhamnoside and enzymes with scores lower than those of all positive drugs. Fig 12 further provided additional annotations to the molecular docking figures of these compounds. The results showed that these active ingredients in the extracts were able to form strong interactions with individual enzyme residues and the detailed information was shown in the Table 6. Compound 33 (Quercetin 3-O-galactoside 7-O-rhamnoside) was more potent against the four enzymes compared to the positive drugs. To a certain extent, it could indicate that SPPE was a candidate drug for hypoglycemic, whitening and anti-Alzheimer’s disease.

**Table 6:** The output data of molecular docking report.

Compound	Score	Binding residues bases	Binding residues	Linkages types	E(kcal/mol)	mode
Acarbose	-13.9966	2	GLN	H-acceptor	-1.0	(1)
			LYS	H-acceptor	-4.8	
Quercetin 3-O-galactoside 7-O-rhamnoside	-16.5369	6	TRP	H-donor	-0.8	(2)
			GLU	H-donor	-1.1	
			ASP	H-donor	-3.8	
			HIS	H-donor	-1.0	
			ASP	H-donor	-1.1	
			ILE	H-acceptor	-0.2	
Acarbose	-13.9128	5	LEU	H-donor	-1.0	(3)
			ASP	H-donor	-1.4	
			VAL	H-donor	-1.3	
			PHE	H-acceptor	-2.5	
			VAL	H-acceptor	-0.8	
Quercetin 3-O-galactoside 7-O-rhamnoside	-16.9190	4	PHE	H-donor	-1.7	(4)
			LEU	H-donor	-1.4	
			ALA	H-donor	-1.0	
			PHE	H-acceptor	-3.1	
Kojic acid	-10.1190	2	ASN	H-honor	-0.8	(5)
			THR	H-honor	-1.7	
Quercetin 3-O-galactoside 7-O-rhamnoside	-16.7917	2	GLN	H-donor	-1.9	(6)
			ILE	Pi-H	-0.6	
Donepezil	-15.6758	1	TYR	H-accept	-1.7	(7)
Quercetin 3-O-galactoside 7-O-rhamnoside	-25.8473	5	TRP	H-donor	-1.8	(8)
			GLY	H-donor	0.3	
			TYR	H-donor	-0.8	
			ILE	H-donor	-3.0	
			TYR	H-acceptor	-1.3	

## 4. Discussion

The extraction and purification of seaweed polyphenols is a highly challenging task, as the polyphenol content in most seaweeds is subject to fluctuations that are influenced by environmental conditions and intrinsic factors [10]. The most traditional and widely used extraction method is solvent extraction, which is technically more mature and does not require specialized equipment. Nevertheless, the prevalence of numerous organic solvents with high toxicity, low extraction rates, and challenges in separation and purification has led to a shift towards emerging technologies such as ultrasonic extraction, microwave extraction, supercritical fluid extraction, and pressurized hydrothermal extraction. In this study, ultrasonic-assisted extraction was employed due to its ability to shorten extraction time, reduce energy consumption, and enhance extraction efficiency.

In this study, the optimal extraction process was obtained through single-factor investigation and orthogonal analysis, including an ethanol concentration of 50%, ultrasonic time of 40 minutes, a solid-liquid ratio of 1:34 (g/mL), a temperature of 65°C, and two extraction cycles. Fig 1 showed that the extraction rate of polyphenols increased first and then decreased with the increase of ethanol concentration, and the optimal extraction effect was achieved when the ethanol concentration was 50%. Due to the high concentration of ethanol, its polarity was very different from that of polyphenol components. As a result, the solubility of polyphenols decreased in an ethanol solution. Additionally, pigments, alcohol-soluble impurities, and highly lipophilic components could also be dissolved. It might also be that high concentration of ethanol disrupted the hydrogen bonds and hydrophobic interactions within polyphenols. Therefore, an ethanol concentration that was too high or too low could result in the decomposition of polyphenols and expansion of tissues, which was unfavorable. In addition, previous studies also showed that the optimal extraction condition for polyphenols was 50% ethanol concentration [9]. Furthermore, a suitable high temperature could accelerate the dissolution and diffusion of polyphenols, thereby increasing the extraction yield of polyphenols [43–45]. Above 60°C, the extraction rate began to decline. This may be attributed to the high temperature causing the degradation of polyphenols, as well as the volatilization of solvents at high temperatures, which was not conducive to the dispersion of polyphenols. After conducting single-factor investigation, orthogonal test and related studies, the optimal extraction temperature was 65°C. High-purity polyphenols from *Sargassum pallidum* were obtained by purification using macroporous resins LS-305A, Sephadex LH-20 and ethanol elution. This study confirmed that the combination of macroporous resin and Sephadex could obtain high-purity algal polyphenols.

After conducting a literature search, we fixed an ultrasonic power that had minimal impact on the polyphenol extraction rate. Although the final extraction yield might be higher, it was possible to obtain better results by investigating this factor during the experiment. Additionally, ultrasound can cause temperature increases and oxidative reactions, which may lead to the degradation or loss of polyphenols. Exploring various methods or techniques in conjunction with UAE is advisable to enhance extraction efficiency and stability, such as solvent extraction, supercritical fluid extraction (SFE), microwave-assisted extraction (MAE), and pressurized liquid extraction (PHLE).

Based on their chemical structure, natural polyphenols can be classified into flavonoids, phenolic acids, lignans, stilbenes and other polyphenols [46]. All of the polyphenol compounds including 7 organic acids, 8 phenols and 19 flavonoids in Table 5 were first identified in SPPE. Among the eight phenols, all of these compounds possed at least one aromatic phenolic ring and one or more highly polymerizable hydroxyl substituents.

Furthermore, based on the compounds identified by UPLC-QTOF-MS/MS, molecular docking analysis revealed that Quercetin 3-O-galactoside 7-O-rhamnoside in SPPE could form hydrogen bonds and interact with enzymes, displaying stronger binding affinity to the enzymes than the positive drugs. This suggested a higher potential for Quercetin 3-O-galactoside 7-O-rhamnoside as an enzyme inhibitor. However, this study did not isolate and test each potential active ingredient individually. Future research using cell or animal models is necessary to further elucidate their activities.

## 5. Conclusion

The extraction and purification techniques of polyphenols were optimized in this work, with the ideal extraction parameters being an ethanol concentration of 50%, an extraction duration of 40 minutes, an extraction temperature of 65°C, and two extraction cycles. An extract with a high polyphenol content (7.5 mg GAE/g) and purity (70.5%) was successfully obtained. A total of 50 compounds were characterized in SPPE and enzyme inhibition assay showed that SPPE had significant potential hypoglycaemic, whitening and anti-Alzheimer’s effects. Molecular docking analysis further supported these findings, highlighting the potential of Quercetin 3-O-galactoside 7-O-rhamnoside as a strong enzyme inhibitor.

Furthermore, the mechanism of the enzyme inhibitory effect on the active site remained to be fully understood and required further investigation. Additionally, the hypoglycemic, whitening, and anti-Alzheimer’s activities of the extract in vivo, as well as the inhibitory mechanism of action, should be further studied and characterized.

## Supporting information

S1 Data (RAR)
